# Lack of relationship between Alu repetitive elements in angiotensin converting enzyme and the severity of diabetic retinopathy

**DOI:** 10.5937/jomb0-27885

**Published:** 2021-06-05

**Authors:** Abu-Hassan Diala Walid, Muawyah D. Al-Bdour, Mohammed El-Khateeb

**Affiliations:** 1 University of Jordan, School of Medicine, Department of Physiology and Biochemistry, Amman, Jordan; 2 University of Jordan, School of Medicine, Department of Ophthalmology, Amman, Jordan; 3 Jordan University Hospital, Amman, Jordan + National Center for Diabetes, Endocrinology and Genetics, Amman, Jordan; 4 National Center for Diabetes, Endocrinology and Genetics, Amman, Jordan

**Keywords:** angiogenesis, complications, diabetes, polymorphism, retina, angiogeneza, komplikacije, dijabetes, polimorfizam, mrežnjača

## Abstract

**Background:**

Angiotensin-converting enzyme (ACE) stimulates angiogenesis that leads to the development of diabetic retinopathy (DR). Alu repetitive elements in ACE gene increase the expression of this enzyme. We investigated the frequency of Alu repetitive elements, insertion/deletion (I/D) polymorphism, in angiotensin-converting enzyme among diabetic retinopathy patients and whether this polymorphism is associated with the severity of retinopathy in Jordanians with type 2 diabetes.

**Methods:**

A total of 277 subjects participated in this case/ control study (100 diabetic patients without DR, 82 diabetic patients with DR, and 95 healthy control). Blood samples were withdrawn, followed by DNA extraction. Alu repetitive elements were examined by polymerase chain reaction followed by gel electrophoresis.

**Results:**

The genotype and allele frequencies among diabetic patients, were close to healthy controls (genotypes, II 44.4 vs. 44.7%, ID 44.4 vs. 42.6%, DD 12.2 vs. 12.8%, P = 0.402 and 0.677 respectively, alleles, I 65.6 vs. 66%, D 34.4 vs. 34%, P=0.863). Complicated diabetics with retinopathy showed similar genotype and allele frequency to those without complications. The severity of diabetic retinopathy in affected individuals was not correlated with I/D polymorphism (P=0.862).

**Conclusions:**

We conclude that the presence of Alu repetitive elements did not increase the development or progression risk to retinopathy in Jordanian type 2 diabetic patients. No association between I or D alleles with the severity of DR was detected.

## Abstract (sr)

**Uvod:**

Anagiotenzin konvertujući enzim (ACE) stimuliše angio genezu koja dovodi do razvoja dijabetičke retinopatije (DR). Alu repetitivni elementi u genu ACE povećavaju ekspresiju ovog enzima. Istražili smo učestalost Alu ponavljajućih elemenata, polimorfizam insercije/delecije (I/D) u angiotenzin konvertujućem enzimu među pacijentima sa dijabetičnom retinopatijom i da li je ovaj polimorfizam povezan sa ozbiljnošću retinopatije kod Jordanaca sa dijabetesom tipa 2.

**Metode:**

U ovoj studiji sa kontrolnom grupom učestvovalo je ukupno 277 ispitanika (100 pacijenata sa dijabetesom bez DR, 82 pacijenta sa dijabetesom sa DR i 95 zdravih pacijenata). Uzeti su uzorci krvi, nakon čega je izvršena ekstrakcija DNK. Alu repetitivni elementi su ispitivani lančanom reakcijom polimeraze praćenom gel elektroforezom.

**Rezultati:**

Učestalost genotipa i alela kod dijabetičara bila je blizu zdravih kontrolnih pacijenata (genotipovi, II 44,4 naspram 44,7%, ID 44,4 naspram 42,6%, DD 12,2 naspram 12,8%, P = 0,402 i 0,677, aleli, I 65,6 naspram 66%, D 34,4 nasuprot 34%, P = 0,863, respektivno). Komplikovani dijabetičari sa retinopatijom pokazali su sličan genotip i frekvenciju alela onima bez komplikacija. Ozbiljnost dijabetičke retinopatije kod pacijenata nije u korelaciji sa I/D polimorfizmom (P = 0,862).

**Zaključak:**

Zaključili smo da prisustvo ponavljajućih elemenata Alu repetitivnih elemenata nije povećalo rizik od razvoja ili progresije retinopatije kod jordanskih pacijenata sa dijabetesom tipa 2. Nije otkrivena povezanost alela I ili D sa težinom DR.

## Introduction

### Retinopathy as a diabetic complication

Diabetic retinopathy (DR) is a microangiopathic complication in patients affected by diabetes mellitus [1] [2] [3] [4]. As a common finding in diabetic patients and an essential global cause of blindness in working-age individuals, several risk factors have been closely correlated with the development and progression of DR complication, including blood glucose levels, the type of diabetes, duration of disease, blood pressure, and possibly lipid profile [1] [2] [3] [4]. The histopathological features include loss of pericytes, basement membrane hypertrophy, microaneurysm formation, formation of blood vessels or neovascularization, capillary occlusions, enhanced vascular permeability, and fibrovascular proliferation [1] [2] [3] [4]. The primary cause of vision loss in diabetic patients is mainly a result of intraocular angiogenesis, which leads to proliferative diabetic retinopathy (PDR), and leakage of retinal vessels, which leads to diabetic macular edema (DME) [5]. Many studies have investigated and established a correlational relationship between chronic hyperglycemia and the development of DR; nonetheless, the mechanism by which hyperglycemia results in damaging retinal microvasculature is still unclear.

### Angiotensin-converting enzyme (ACE)

ACE converts angiotensin I to angiotensin II that acts as an active vasoconstrictor. Angiotensin II upregulates the expression of vascular endothelial growth factor (VEGF), resulting in the induction of angiogenesis [6]. Stimulation of angiogenesis has a role in the development of diabetic retinopathy [6].

### Alu elements in the human genome

Alu elements are primate-specific mobile elements that are found in more than a million copies in the human genome, comprising 11% of it [7]. They continue to insert in the modern human genome, resulting in genetic diversity and contributing to disease development through insertional mutagenesis and non-allelic homologous recombination events that cause copy number variation. Alu elements are ubiquitously present in many genes influencing their expression through their effects on polyadenylation [8] [9], splicing [10] [11] [12], and ADAR (adenosine deaminase that acts on RNA) editing [13] [14] [15].

### ACE polymorphisms and DR

Polymorphisms in various genes have been found in patients with DR, including an Alu insertion element, insertion/deletion (I/D) polymorphism in* ACE* gene [16] [17]. This polymorphism corresponds to an Alu repetitive sequence of 287 bp inserted in intron 16 [16] [17]. The Alu repetitive sequence is found in three forms: D/D and I/I homozygotes and I/D heterozygotes. D refers to the deletion of the Alu element, whereas I refers to the insertion of this element. The Alu element increases *ACE* promoter transcriptional activity by about 70% leading to an elevation of the level of ACE in serum. ACE levels were found elevated in type 2 diabetic patients, particularly in patients with retinopathy [18] [19]. Studies on different populations that tested the *ACE* I/D polymorphism in DR patients showed both association and no relationship between this polymorphism and DR [20] [21] [22] [23] [24] [25] [26] [27] [28] [29] [30] [31].

In this study, our aim is to determine the prevalence of the insertion DNA polymorphism detected in the *ACE* gene in Jordanian type 2 diabetic patients and the relationship between this polymorphism and DR severity.

## Materials and Methods

### Patient selection and sampling

In this cross-sectional study, 277 subjects were recruited from the Ophthalmology clinics at the National Center for Diabetes, Endocrinology and Genetics (NCDEG). Subjects were grouped into 3 categories; diabetic with retinopathy (82 patients), diabetic without retinopathy (100 patients), and healthy controls (95 individuals). Subjects attending the ophthalmology clinic at NCDEG who matched the following selection criteria were recruited in this study.

Inclusion criteria included: age ranges between 28 to 88 years, type 2 diabetes diagnosed by standard means defined by the American Diabetes Association, diabetes disease duration less than 15 years with no retinal problems prior to the diagnosis of diabetes. The American Diabetes Association standards for diagnosing diabetes include using one of four tests to establish a firm diagnosis of diabetes: (i) fasting plasma glucose (FPG) > 6.9 mmol/L (> 125 mg/dL), most commonly used test; (ii) Random plasma glucose ≥11.1 mmol/L (200 mg/dL) with diabetes symptoms such as polyuria, polydipsia, fatigue, or weight loss; (iii) two-hour post-load glucose ≥1 1.1 mmol/L (≥ 200 mg/dL) on a 75 g oral glucose tolerance test; (iv) or HbA1c ≥ 48 mmol/mol (≥ 6.5%). All these tests require confirmation with a second test, which may be the same test or a different test. The controls were sampled from the general population to which the cases belonged with no history of diabetes as reported by subjects. HbA1c test was performed for diabetic patients, whereas patients' record of the absence of diabetes was the reference for controls. Demographic data, such as age, gender, weight, and height, were collected. Medical history of diabetes, hypertension, ischemic heart disease, and dyslipidemia was obtained. Consistent gender distribution in each group was also considered. The following characteristic changes were considered to detect retinopathy: new vessels, hemorrhages, exudates, and fibrous proliferation. These changes were examined by slitlamp biomicroscopy through dilated pupils by an experienced ophthalmologist. DR severity was determined based on the evidence-based International Clinical DR Disease Severity Scale that is agreed on by the American Academy of Ophthalmology (AAO) in 2001 and the International Council of Ophthalmology (ICO) in 2002. Blood samples were collected in EDTA tubes for DNA extraction.

### Ethical approval

This project was conducted after the institutional and national formal approvals of the Deanship of Scientific Research at the University of Jordan, and the Institutional Review Board (IRB) at the NCDEG were obtained. Written informed consent was obtained from all individual participants included in the study before a blood sample was withdrawn. Each subject was given a coding number to preserve privacy rights. The study was performed in compliance with the ethical principles and standards outlined in the Declaration of Helsinki and the Code of Ethics of the World Medical Association for experiments involving humans.

### DNA extraction, polymerase chain reaction (PCR) and gel electrophoresis

Samples were handled according to published protocols. DNA extraction was performed from whole blood using QIAGEN Puregene Blood Core Kit B (QIAGEN Sciences, Maryland, USA) according to the manufacturer's instructions. DNA purity was verified by measuring the absorbance ratio at 260 nm/280 nm using Biochrom™ Lightwave spectrophotometer, UK. A ratio of 1.8-2 was accepted. DNA concentration was measured by exposing samples to ultraviolet light at 260 nm in the aforementioned spectrophotometer, followed by measuring the optical density and the automatic calculation of the concentration by the device. The insertion/deletion DNA polymorphism was detected in intron 16 of the human ACE gene by semi-quantitative PCR. The forward primer sequence was CTGGAGACCACTCCCATCCTTCT, and the reverse primer was GATGTGGCCATCA-CATTCGTCAGAT. The PCR conditions were: 5 minutes of initial denaturation at 94°C, followed by 30 Cycles of 94°C for 60 seconds, 58°C for 60 seconds, and 72°C for 120 seconds, with a final extension at 72°C for 7 minutes using Bio-Rad, S1000 Thermal Cycler™, USA. PCR products were detected on a 2% agarose gel. Quality control for genotyping was ensured by running a negative control that contains all PCR components except the DNA template in every PCR run, repeating around 16% of all samples by different lab personnel, and testing for Hardy Weinberg (HW) equilibrium.

### Genotyping

The insertion corresponds to an Alu repetitive sequence and is 287 bp long. The detected fragment sizes were: 190 bp band for normal genotype (DD), 490 bp band for homozygous (II), and 190 and 490 bp bands for heterozygous (ID).

### Statistical analysis

Statistical Package for Social Sciences (SPSS) version 16 was used to perform statistical analysis (Chicago, Illinois, USA). All values represent mean ± standard deviation (SD), or counts (%). The correlation of DR with quantitative variables, such as HbA1c levels, was detected by analysis of variance (ANOVA), whereas logistic regression was used to evaluate whether a categorical variable, such as genotype, is correlated with another variable. Quantitative results were shown as mean ± standard deviation. Genotype and allele frequencies between different groups were analyzed by the Chi-square χ^2^ test. Geno type and allele frequency were analyzed for concordance to the Hardy-Weinberg equilibrium. Results were considered statistically significant when P-value is less than 0.05.

## Results

The clinical characteristics of the subjects in each of the three groups were shown in Table 1. No significant difference between them in age or gender was detected. Hypertension (HTN) and dyslipidemia (DLP) were significantly more prevalent in diabetic patients than in healthy controls – Table 1. HbA_1C_ was not significantly different between diabetic patients with DR or without it – Table 1. The duration of diabetes in diabetic patients with DR was significantly longer than in diabetic patients without DR – Table 1.

**Table 1:** Clinical characteristics of normal controls and diabetic patients with or without complicating retinopathy BMI, body mass index; DM, diabetes mellitus; DLP, dyslipidemia; HTN, hypertension; IHD, ischemic heart disease; UA, unavailable; NA, not applicable. Qualitative results are shown as percentages. Quantitative results are shown as mean + standard deviation. *P-value compares people with diabetes with DR to people with diabetes without DR and healthy controls. Clinical characteristics of subjects in the three groups (diabetic patients, with or without diabetic retinopathy, and controls). Gender distribution was shown in counts. The average + standard deviation was calculated for other parameters, including age, BMI, HbA1C, and duration of diabetes mellitus. The percentages of subjects with hypertension, ischemic heart disease, and dyslipidemia were calculated. A P-value of less than 0.05 was considered statistically significant.

Group	Clinical Characteristics							
	Gender (M:F)	Age (years)	BMI (kg/m^2^)	HTN (%)	IHD (%)	DLP (%)	HbA_1C_ (%)	Duration of DM (years)
Control (n=95)	59:36	55 ± 13	27.7 ± 4.6	20.8	5.7	19.8	UA	NA
DM without DR (n=100)	39:61	60 ± 8	31.5 ± 6.2	67.0	21.0	78.0	7.7 ± 1.1	7.0 ± 4.3
DM with DR (n=82)	50:50	62 ± 8	31.9 ± 5.7	71.6	17.3	67.1	7.7 ± 1.1	10.8 ± 4.1
*P	0.969	0.690	0.800	0.000	0.363	0.004	0.834	0.0001

Table 2 represented the distribution of *ACE* genotypes and allele frequencies among diabetic patients, combining both groups with and without retinopathy (the genotypes of 180 of 182 participants were obtained due to sample depletion or failure of PCR), and controls (the genotypes of 94 of 95 participants were obtained). Genotype distribution was similar, with no significant differences between diabetic patients and healthy controls in allele and genotype frequency – Table 2. In Table 3 we compared diabetic patients without the complicating retinopathy to their counterparts with retinopathy. ID genotype was the most common among retinopathy-complicated patients but not significantly – Table 3. Additionally, the allele frequency was very close to each other in the groups with or without retinopathy – Table 3. The allelic distribution of the I/D polymorphism was in Hardy Weinberg equilibrium (χ^2^: 0.288, P=0.866). We also grouped DD and ID genotypes of people with diabetes without DR and compared them to DD of people with diabetes and DR and found a significant association between retinopathy and DD genotype in DR patients when compared to non-risk DD and ID genotypes in diabetic patients without DR, P=0.002.

**Table 2:** Distribution of Alu repetitive elements genotype and allele frequencies among control and diabetic patients Percentages are shown in parenthesis. Alu repetitive elements genotype and allele frequency in diabetic patients and healthy control subjects. Counts and percentages of II, ID, and DD genotype in addition to I and D allele were calculated. A P-value of less than 0.05 was considered statistically significant.

Categories	Controls (n=94)	Diabetic Cases (n=180)	P
Genotype			
II	42 (44.7)	78 (43.3)	
ID	40 (42.6)	80 (44.4)	0.402
DD	12 (12.8)	22 (12.2)	0.677
Alleles			
I	124 (66.0)	236 (65.6)	
D	61 (34.0)	124 (34.4)	0.863

**Table 3:** Distribution of Alu repetitive elements genotype and allele frequencies among diabetic patients with or without retinopathy Percentages are shown in parenthesis. Alu repetitive elements genotype and allele frequency in diabetic patients with or without diabetic retinopathy. Counts and percentages of II, ID, and DD genotype in addition to I and D allele were calculated. A P-value of less than 0.05 was considered statistically significant.

Categories	Diabetic Cases without Retinopathy (n=100)	Diabetic Cases with Retinopathy (n=80)	P
Genotype			
II	48 (48.0)	30 (37.4)	
ID	39 (39.0)	41 (51.3)	0.538
DD	13 (13.0)	9 (11.3)	0.870
Alleles			
I	135 (67.5)	101 (63.1)	
D	65 (32.5)	59 (36.9)	0.682

We correlated the clinical characteristics of diabetic patients who developed retinopathy with *ACE* genotypes in Table 4. No significant differences between patients with any of the genotypes in age (P=0.272), BMI (P=0.445), HbA1C (P=0.550), or duration of diabetes (P=0.537) were detected. The presence of one copy or both copies of the D allele was not significantly associated with proliferative DR (P_ID vs. II_ was 0.510, and P_DD vs. II_ was 0.348). Our results did not show a significant effect of this polymorphism on the severity of DR (P=0.862) – Table 5.

**Table 4:** Clinical characteristics of diabetic patients according to Alu repetitive elements genotypes BMI, body mass index. Data are represented as means + SD. Percentages are shown in parenthesis. *P-value compares II to ID and DD. Clinical characteristics of diabetic patients, with or without diabetes, and their relationship to Alu Repetitive Elements Genotype. Gender distribution was shown in counts. The average + standard deviation was calculated for other parameters, including age, BMI, HbA1C, and duration of diabetes mellitus. The counts and percentages of subjects with hypertension, ischemic heart disease, and dyslipidemia were calculated. A P-value of less than 0.05 was considered statistically significant.

Characteristic	II (n=30)	ID (n=41)	DD (n=9)	*P
Gender	14:16	23:18	3:6	0.707
Age (years)	62 ± 7	62 ± 10	62 ± 8	0.907
BMI (kg/m^2^)	32.1 ± 6.7	32.1 ± 5.1	32.4 ± 4.4	0.927
Hypertension	20 (66.7)	29 (70.7)	6 (66.7)	0.556
Ischemic	5 (16.6)	7 (17.1)	2 (22.2)	0.816
Dyslipidemia	22 (73.3)	27 (65.9)	4 (44.4)	0.355
HbA1C	7.6 ± 1.2	8.0 ± 1.1	7.2 ± 1.0	0.425
Duration of	11.1 ± 4.2	10.5 ± 3.9	9.4 ± 4.6	0.385

**Table 5:** Alu repetitive elements genotypes and the severity of diabetic retinopathy Data are represented as percentages. The relationship of Alu repetitive elements genotype in patients with diabetic retinopathy and the severity of the disease. Frequencies and percentages of II, ID, and DD genotype in relation to the severity of retinopathy were calculated. A P-value of less than 0.05 was considered statistically significant.

Retinopathy Severity	II (n=30) %	ID (n=41) %	DD (n=9) %
Mild	26.7	29.3	11.1
Moderate	30.0	31.7	22.2
Severe	23.3	15.0	33.3
Proliferative	20.0	25.0	33.3

The Alu element polymorphism did not affect the age of onset of diabetes in patients with or without DR (P 0.198). The onset of diabetes did not show a statistically significant correlation with the severity of DR (P 0.170). In reference to patients' records, we found that the severity of DR in affected subjects was significantly associated with the presence of other ophthalmic problems including, but not limited to, cataract, glaucoma, and acute macular degeneration (P 0.0005). Additionally, we determined whether there was gender variability in genotype distribution and found that the homozygous DD genotype was more than twice as common in females as in males with diabetic retinopathy; however, the distribution of Alu genotypes according to gender was not significantly different (P=0.467).

## Discussion

The D allele of *ACE* gene is more frequent in the Arab populations, including Jordanians (0.66), compared to other populations [32]. It has a higher frequency among sub-Saharan Africans [33] and Arabs (0.60-0.76) [34] [35] than in Caucasians (0.46-0.51), Asian populations (0.29-0.46) [33] [36], and Yanomami Indians, Samoans, and Australian Aborigines (0.15, 0.09 and 0.03, respectively) [33] [37]. The frequency of the *ACE *I/D polymorphism genotypes varies between races. In our study sample, ID (43.8%) and II (43.8%) genotypes were more frequent than DD genotype (12.4%). Alu insertion polymorphisms, such as *ACE* I/D polymorphism, are appropriate for investigating genetic variation in the human population due to several properties. Firstly, they are stable markers since they result only from an insertion event of an Alu element into a new chromosomal location; therefore, the ancestral genotype is defined as the absence of insertion at a specific chromosomal position. Secondly, they are easy to detect by PCR amplification and gel electrophoresis. In this study, we did not show an association between I/D polymorphism and the risk of developing diabetic retinopathy. Both allele and genotype frequency did not significantly associate with DR when comparing diabetic patients to controls or diabetic patients with DR to those without it. Based on the staging and severity of retinopathy, subgroup analysis did not show a significant association of I/D polymorphism in *ACE* gene with the incidence or progression at early or advanced stages of DR. However, our study was the first one that investigated the frequency of the *ACE* gene I/D polymorphism among Jordanian individuals with diabetic retinopathy. Studies by other groups showed that the deletion in the *ACE* gene was associated with the prevalence of proliferativeretinopathy in type 1 diabetic patients suggesting that the DD genotype may increase the risk of proliferative retinopathy [26]. The DD genotype was significantly more common in Iranian type 2 diabetic patients with DR than those without DR [27]. In the Pakistani population, the *ACE* I/D polymorphism showed a significant association with DR and the mild form of the disease, non-proliferative DR (NPDR), but not with the severe type of the disease, PDR [28]. In another study involving the Pakistani population, male patients exhibited a significant association between DR stages and* ACE* I/D polymorphism [29]. A significant relationship between D allele polymorphism in the *ACE* gene and advanced DR was reported in Japanese individuals with type 2 diabetes [24]. Other studies examined the association of *ACE* I/D polymorphism and DR but found no association, including a meta-analysis that involved 2,342 cases with DR, both type 1 and type 2 diabetic patients, and 2,048 healthy controls [38]. Another meta-analysis of six studies on type 1 diabetic patients with I/D polymorphism and seven studies on type 2 diabetic patients with this polymorphism suggested a non-significant association of this polymorphism and the development of any type of DR [32]. Wide differences in the frequency of *ACE* genotypes and their correlation with this disease when comparing different populations highlight the great care that should be taken when dealing with clinical data that associate *ACE* alleles with different diseases. Although the justification for conflicting results is not yet obvious, the possible reasons for such controversy are the racial differences of the subject populations, variable sample size in different studies, and differences in sampling methods. Due to the inconsistencies in the findings related to the clinical significance of the *ACE* genetic polymorphisms in different populations, additional studies should be performed to con firm the relationship between *ACE* gene and retino pathy in different ethnic populations. Additionally, the role of other possible contributing regulatory element of *AC* *E* or another gene near* ACE* and the effect of haplotypes of gene variants should be investigated. The results we obtained need confirmation by larger sample size, and prospective and familybased studies, studies on different ethnic groups, functional studies, and long-term follow-up studies. In addition to that, mechanistic studies investigating the possible pathophysiological mechanisms through which D allele mediates the pathogenesis of DR particularly advanced stages of this complication, should be performed in the future.

The limitations of this study included the relatively small sample size, a wide age range of subjects, and the difficulty of matching cases and controls in relation to some variables such as disease duration. In addition, healthy subjects (controls) were not tested to confirm that they were free of diabetes or retinal diseases at the time of recruitment, nor were followup tests performed later. DNA sequencing could have been done to confirm results, but financial limitations interfered with performing sequencing. However, to the best of our knowledge, our study is the first study of this *ACE* polymorphism in Jordanian diabetic patients. Our study compared diabetic patients to normal control healthy individuals in addition to the comparison of diabetic retinopathy patients to diabetic patients without DR. It also subdivided the severity level of the non-proliferative type of DR into several ones. Overall, case-control association studies can be helpful in the identification of disease biomarkers and the analysis of multiple potential factors for complex diseases such as DR.

In conclusion,* ACE* I/D polymorphism in the renin-angiotensin system did not show a potential influence on the development or progression of DR in the Jordanian population, as shown in this study. However, we were able to determine the frequency of *ACE* I/D polymorphism in diabetic patients with and without retinopathy. Although we did not find a correlation between DR and this polymorphism, these results might be helpful to conclude that the assessment of the *ACE* I/D polymorphism may not be a reliable tool in identifying patients at risk to develop diabetic retinopathy or those with the suspected severe complication, at least in Jordanians.

## Acknowledgments

We thank the Deanship of Scientific Research at the University of Jordan for funding this project. We also thank the National Center for Diabetes, Endocrinology and Genetics for facilitating patient recruitment and data collection.

## Funding

The Deanship of Scientific Research, the University of Jordan, Amman, Jordan 11942 (grant number 19/2015/2521). The sponsor only reviewed the proposal of this project and had no role in the design of the study, data collection, data analysis, interpretation of data, or manuscript writing.

## Conflict of interest statement

The authors stated that they have no conflicts of interest regarding the publication of this article.

## List of abbreviations

AAO, American Academy of Ophthalmology; ADAR, adenosine deaminase that acts on RNA; ANOVA, Analysis of variance; ACE, angiotensin-converting enzyme; BMI, body mass index; DME, diabetic macular edema; DR, diabetic retinopathy; HW, Hardy Weinberg; ICO, International Council of Ophthalmology; NCDEG, National Center for Diabetes, Endocrinology and Genetics; PCR, polymerase chain reaction; PDR, proliferative diabetic retinopathy; RAS, renin-angiotensin system; SD, standard deviation; VEGF, vascular endothelial growth factor.
